# Informed consent in critically ill adults participating to a randomized trial

**DOI:** 10.1002/brb3.1965

**Published:** 2020-12-03

**Authors:** Milène Guinchard, Loane Warpelin‐Decrausaz, Kaspar Schindler, Stephan Rüegg, Mauro Oddo, Jan Novy, Vincent Alvarez, Andrea O. Rossetti

**Affiliations:** ^1^ Department of Clinical Neuroscience Lausanne University Hospital and University of Lausanne Lausanne Switzerland; ^2^ Clinical Trial Unit Lausanne University Hospital and University of Lausanne Lausanne Switzerland; ^3^ Sleep‐Wake‐Epilepsy‐Center, Department of Neurology Inselspital, Bern University Hospital, University of Bern Bern Switzerland; ^4^ Department of Neurology University Hospital Basel and University of Basel Basel Switzerland; ^5^ Department of Intensive Care Medicine Lausanne University Hospital and University of Lausanne Lausanne Switzerland; ^6^ Department of Neurology Hôpital du Valais Sion Switzerland

**Keywords:** Declaration of Helsinki, electroencephalography, Ethic Commission, IRB, Switzerland

## Abstract

**Objective:**

The 2014 update of the Swiss law on research increases patients' protection; it adds specific requirements for emergency situations, implying an active search for patients' wishes regarding research participation; the possibility of consent waivers is not clearly stated. We explored its practical impact in a RCT on critically ill adults.

**Methods:**

We considered prospectively collected consents of a multicenter trial addressing the impact of continuous EEG on survival. We assessed the proportions of consents obtained strictly according to the law, of specific waivers for this study obtained from the IRB (early death; relatives' unavailability despite repeated attempts), and the yield of retrieving statements on willingness to research participation. We compared the proportion of consent refusals with those of recent trials in similar environments, and estimated the potential impact on study results.

**Results:**

Of 402 recruited patients, six had double inclusions, one died before intervention, and 27 (6.7%, alive on long‐term) were excluded following consent refusal or withdrawal, leaving 368 analyzable patients. Specific waivers allowed inclusion of 134 (36.4%) patients, while informed consents were obtained for all others. A statement of willingness to research participation was found in only 14.1%. In recent trials, consent refusal oscillated between 0%–23%, according to different waiver policies.

**Conclusions:**

Consent waivers should be specifically foreseen to prevent losing a potentially relevant proportion of patients reaching endpoints, and ensure results generalizability. The yield of looking for willingness to research participation seems low; this questions its current usefulness and calls for a public awareness campaign.

## Graphical abstract

This study analyzes the informed consent flow in a multicenter RCT carried out in critically ill adults and shows that data from 7% of recruited subjects had to be discarded due to lack of consent; this modified the primary endpoint (mortality). Furthermore, 36% of recruited subjects could be analyzed only following a specific waiver accorded from the Ethics Commission. These results highlight critical issues regarding generalizability of results, namely the importance of providing specific waivers in this clinical context and to address drop‐out rates due to lack of consents.

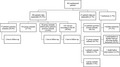

## 1 INTRODUCTION

Obtaining informed consent represents one of the main principles of clinical research enacted by the Declaration of Helsinki (World Medical Association, 2013), Good Clinical Practice (ICH Harmonised Tripartite Guideline E^2016^ (2016), 2016), and by the country's law (in Switzerland: Federal Act on Research involving Human Beings, 2011a). Clinical research on critically ill patients or in emergency situations is essential to attempt decreasing the related morbidity and mortality, but this population cannot be easily involved; specific regulations exist in these settings (Federal Act on Research involving Human Beings, 2011b, 2011c).

In Switzerland, the law regulating research was updated in 2014 (Federal Act on Research involving Human Beings, 2011a). In research with no direct expected benefit, the project must imply “minimal risks,” agreed upon by the Ethic Commission (EC). Moreover, while the possibility to obtain specific consent waivers is not explicitly described (EC may nevertheless grant these, in practice), a patient's statement regarding willingness or opposition to participate to clinical research (made before the lack of capacity) has to be actively sought by investigators, provided there are no “signs and symptoms” showing the patient's unwillingness to participate (Federal Act on Research involving Human Beings, 2011b). This wording appears rather unspecific, especially for critically ill patients. Ideally, a written note should be identified; alternatively, the legal representative may refer a “clear oral statement” by the patient (i.e., the opinion of the legal representative is not relevant). It is questionable if the general population may routinely think at providing such a statement, and if a legal representative may always discriminate between the own opinion and that of the patient. If no patient's opposition is found, investigators in emergency situations should obtain at inclusion a statement by an independent physician with the fiduciary duty of safeguarding patients' interests. Informed consent should be obtained as soon as possible if the patient recovers a capacity of judgment; otherwise, a proxy consent should be sought (Federal Act on Research involving Human Beings, 2011c; Ordinance on Clinical Trials in Human Research, 2013); however, the timeframe of a “permanent lack of judgment capacity” is not clearly defined and thus depends on subjective appreciation.

To our knowledge, application of the current Swiss rules regarding research in emergency situations and patients unable to consent has not been explored; this aspect has received limited attention also in other settings. This work describes the process of informed consent in a trial involving adults with acute consciousness impairment, in order to assess whether current laws can be translated into practice, and identify aspects that may be improved.

## 2 METHODS

### 2.1 Patients and clinical context

Nonconvulsive (subclinical) seizures and status epilepticus (SE) are frequent in comatose patients, and associated with considerable morbidity and mortality (Towne et al., 2000; Zehtabchi et al., 2013). Continuous EEG (cEEG) improves nonconvulsive seizures and SE detection compared with routine EEG (rEEG) lasting <30 min (Claassen et al., 2004) and is broadly recommended in critically ill patients (Claassen et al., 2013; Herman et al., 2015a, 2015b). However, the effect on outcome remains unclear. CERTA (Continuous EEG Randomized Trial in Adults, NCT03129438) (Rossetti et al., 2018) aimed to determine whether cEEG in adults with consciousness impairment correlated with a better outcome than rEEG. It involved four large Swiss hospitals (CHUV Lausanne; Hôpital du Valais; Inselspital Bern; Universitätsspital Basel). Between April 2017 and November 2018, adults with acute consciousness impairment in an intensive/intermediate care unit needing an EEG for clinical purposes were pragmatically recruited and randomized 1:1 to a cEEG (30–48 hr) or 2 rEEG (20 min each; Rossetti et al., 2020; intervention not blinded). The original protocol may be found in the Supporting Information. The primary endpoint was survival at six months. The study was approved by local EC (authorization: 2017‐00268); regulatory procedures were verified by an independent monitor.

### 2.2 Procedures and variables

We retrospectively analyzed the consent procedure of recruited subjects, which occurred under the current Swiss law. Before enrollment, a statement had to be always signed by an independent physician. If the patient recovered judgment capacity, a post hoc consent had to be sought within the 6‐month follow‐up. In the subacute period, if this was impossible after one week (±3 days; defined for this study as the time when judgment capacity was considered “permanently” lacking, considering a compromise between the end of intervention and the need to prevent losing contact with proxy with elapsing time), a proxy consent by a relative or legal representative was sought. During follow‐up at 4 weeks and/or 6 months, investigators contacted the patient, a legal representative, the treating physician, or consulted medical files to evaluate the patient's state (without quantitative cognitive assessments) and obtain a post hoc consent. If consent was refused by proxy or the patient, all collected data had to be discarded. Under predefined conditions, however, in view of the minimal risks related to participation to this RCT felt to be negligible as compared to the potential collective benefit (assessing a biological surveillance but not a therapeutic intervention), waivers specific for this study were obtained from the EC, to allow enrolling patients and using clinical data despite lack of informed consents. This applied in five situations: a—no representative could be identified, or b—despite identification, no consent was collected, despite at least three documented attempts to give information, and 3 others to collect the signature; c—a decision of withdrawal of life‐sustaining therapy was made (to prevent additional distress to the family); d—a patient died before proxy consent was collected (idem); e—an oral agreement was provided to an investigator accompanied by a caregiver witness, unrelated to the study.

Demographical, administrative (statement of wishes, authorization from independent physicians, informed consents obtained or refused/ withdrawn, state of capacity to consent), and clinical information was prospectively collected for the trial. We assessed the proportion of consents obtained in accordance with the law, determined the proportion obtained directly from the patient or from a proxy, and the proportion of patients, proxy, or legal representative consent refusals or withdrawals, stratified for study intervention. We also assessed exceptions in which data were collected according to EC‐granted waivers. We compared the proportion of consent refusals and withdrawals obtained in the CERTA study with six recent large international trials involving critically ill patients (Cooper et al., 2018; Kapur et al., 2019; Lascarrou et al., 2019; Legriel et al., 2016; Navarro et al., 2016; Nielsen et al., 2013). As this is a retrospective analysis of a prospective trial, and Swiss law explicitly states that data from patients who refused participation (even post hoc) should be destroyed, we were unable to explore specific refusal reasons.

### 2.3 Calculations

We present descriptive statistics; frequencies were tested using 2‐sided Fisher's exact tests using STATA version 14.

## 3 RESULTS

**FIGURE 1:**
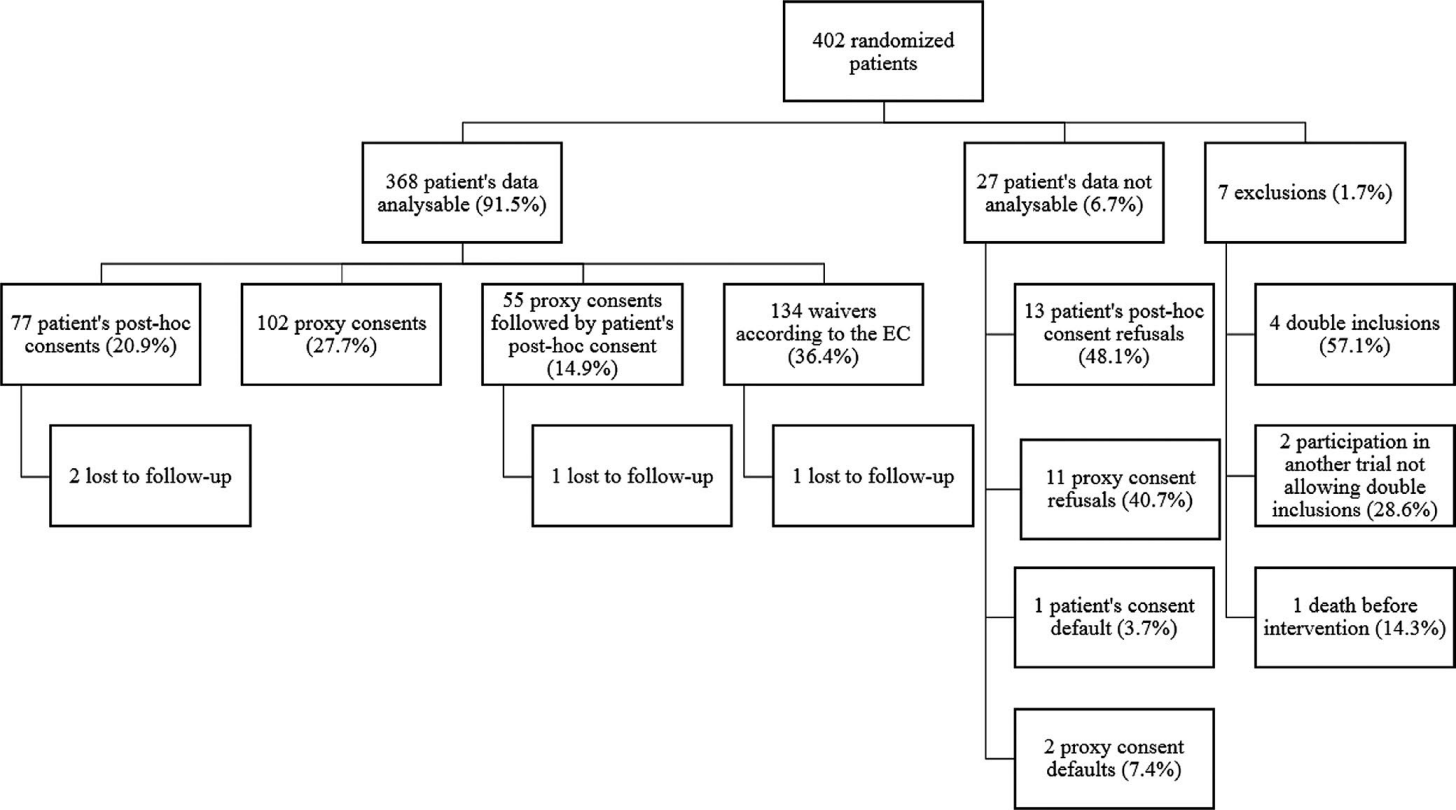
Study participants' flow diagram

The trial included 402 adults with acute consciousness disorders hospitalized in an intensive/intermediate care unit of the four participating hospitals; 201 each received cEEG and rEEG. Seven (1.7%) patients were excluded early (six were included twice, one death prior to intervention), and data from 27 (6.7%) additional participants were unavailable because of consent refusal (24) or lack of it (3; Table 1); there were no differences across centers. Among 24 consent refusals (+3 “consent defaults”: subjects lacking consent and in whom waivers did not apply), 15 (+2) occurred in the rEEG and 9 (+1) in the cEEG arm. We thus had 368 (91.5%) analyzable patients. Four patients were lost to follow‐up, but clinical information was available only until the 4th week (Figure 1).

**TABLE 1:** Distribution of the data among the different sites (column percentages); 7 patients that were excluded early are not reported

	CHUV (318 patients)	Other Swiss sites (84 patients)	*p* (Fisher)
Patients excluded because double inclusions or death before intervention	6 (1.9%)	1 (1.2%)	1.000
Patient's data analyzable	289 (92.6%)	79 (95.2%)	.624
Patient's post hoc consent	61 (19.6%)	16 (19.3%)	
Proxy consent	78 (25.0%)	24 (28.9%)	
Proxy followed by patient's post hoc consent	40 (12.8%)	15 (18.1%)	
Waiver according to the EC	110 (35.3%)	24 (28.9%)	.480
Patient's data not analyzable	23 (7.4%)	4 (4.8%)	.624
Patient's post hoc consent refusal	12 (3.8%)	1 (1.2%)	
Proxy consent refusal	8 (2.6%)	3 (3.2%)	
Patient's consent default	1 (0.3%)	0	
Proxy consent default	2 (0.6%)	0	.576

Note

Consent default means not receiving the consent form from a patient or proxy (who did not decline participation), and impossibility to apply a waiver as defined by the EC.

Abbreviation: EC, Ethics Commission.

The main results are summarized in Figure 1 and Table 1. Written authorizations from an independent physician were collected before inclusion in all 368 analyzable patients. A clear statement regarding willingness to research participation was found in 52/368 (14.1%) of analyzable patients, mostly retrieved orally from a proxy (Table 2) after repeated attempts in an emergency/critical situation. Again, there were no differences across recruiting centers. Of relevance, 134 patients (36.4%) remained in the study and their data were analyzed in the absence of any consent, according to the predefined waivers (Table 1). Data following consent refusals or defaults were destroyed; therefore, we were unable to distinguish patient's post hoc refusal from an oral opposition to participate in the trial from relatives, or from an objection documented in the medical file.

**TABLE 2:** Source of statement of wishes among analyzable patients (column percentages)

	CHUV (289 patients)	Other Swiss centers (79 patients)	*p* (Fisher)
Total per site	38 (13.1%)	14 (17.7%)	0.361
Source			
Relative or legal representative	36 (12.5%)	14 (17.7%)	
Patient's medical file	2 (0.7%)	0	1.000

**FIGURE 2:**
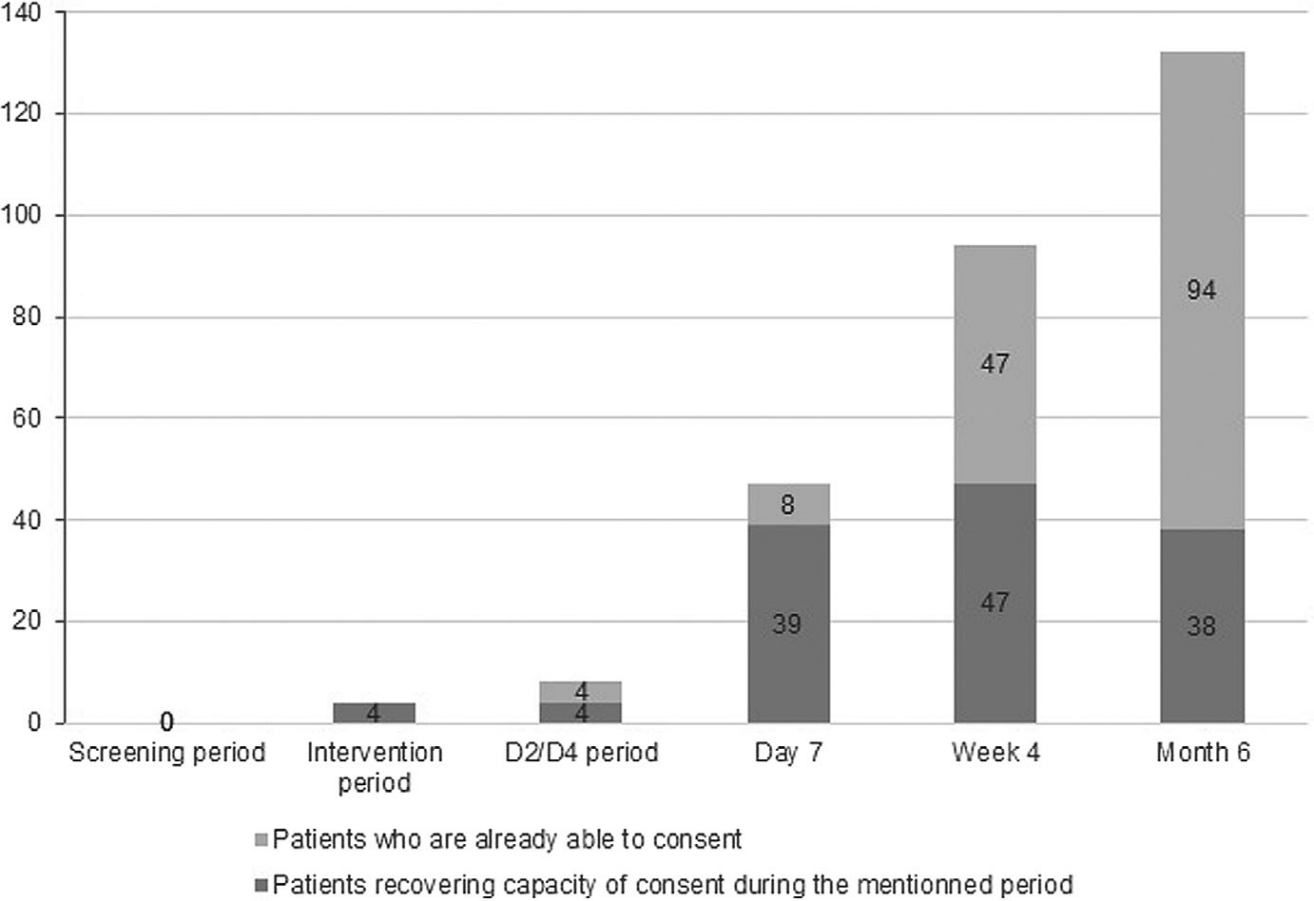
Capacity of consent recovery through the different assessment time points. The number of patients regaining their capacity of consent during the mentioned period is illustrated in dark gray, and the number of patients who already recovered their judgment is in light gray. The whole column represents the total of patients able to consent at the respective time‐point

**FIGURE 3:**
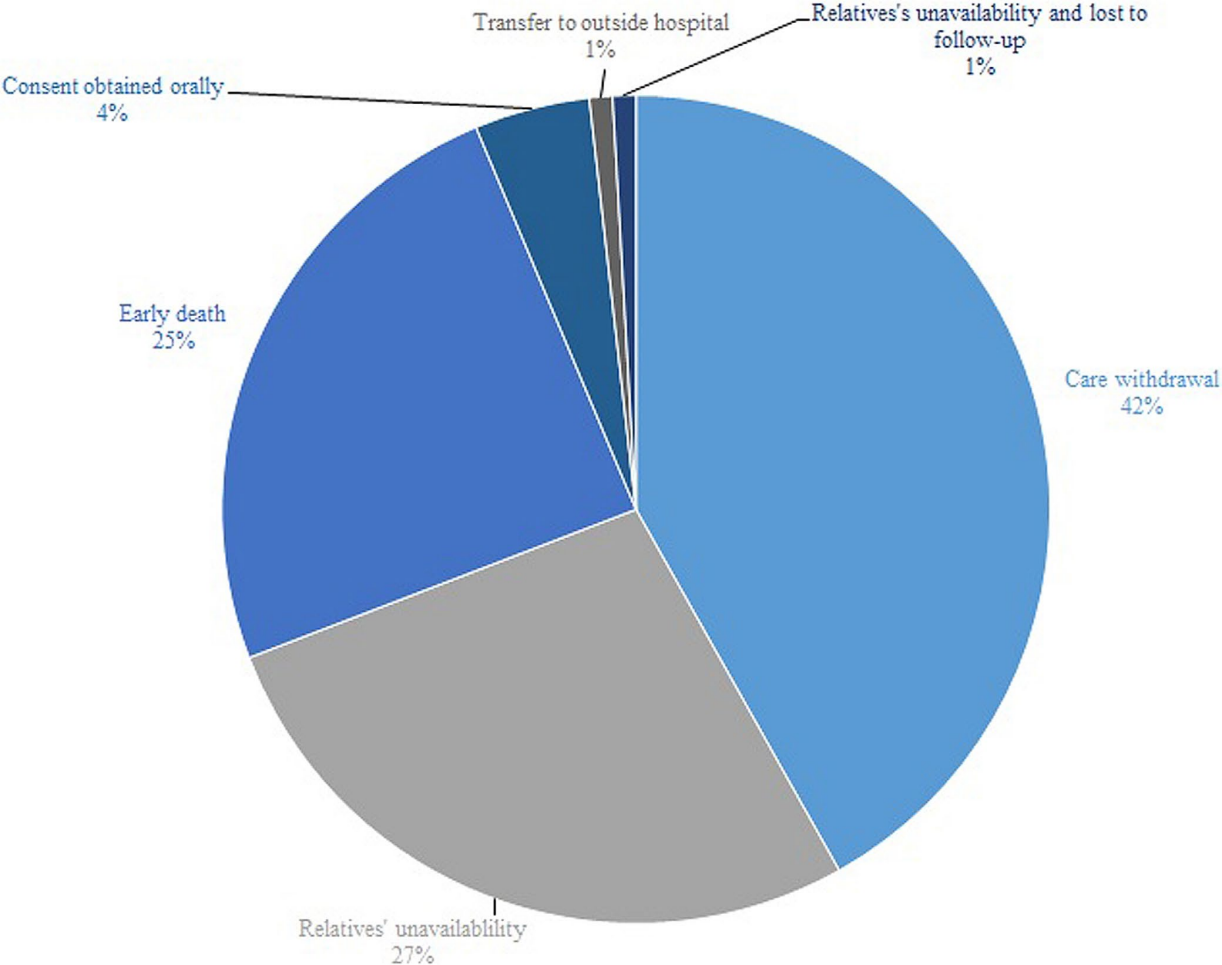
Proportions of the different types of waivers in the absence of informed consent CHUV

Figure 2 illustrates the distribution of recovery of judgment capacity along the study period. In 132 (35.9% of analyzable patients), this occurred between inclusion and the 6 months’ assessment, but for only 8 of them (5.8% of the patients who regained judgment capacity), within the first 4 days, corresponding to the lower limit for the predefined 7 ± 3 days of “permanent lack of judgment capacity.” We further analyzed the type of the 110 waivers collected in the CHUV (details on the waivers in other hospitals were not available; Figure 3): consent was lacking mostly because of care withdrawal or early death. In five cases (4.5%), the form was missing, but a documented, witnessed oral consent was obtained.

Proportions of consent refusals or withdrawals in recent studies involving critically ill patients vary from 0% to 23.2% (Table 3), although detailed information is at times lacking in the papers.

**TABLE 3:** Proportion of consent refusals and withdrawals in various studies

Study	Country	Informed consent process	Study participants	Consent refusals or withdrawals
CERTA (Rossetti et al., 2020)	Switzerland	Enrollment without informed consent, authorization from an independent physician, proxy written informed consent and subsequently from the patient if he/she regained capacity.	402	27 (6.7%)
TTM for Cardiac Arrest with Nonshockable Rhythm (Lascarrou et al., 2019)	France	Participation without informed consent (standard of care), information to relatives only.	548	3 (0.5%)
TTM at 33°C versus 36°C after Cardiac Arrest (Nielsen et al., 2013)	Europe and Australia	Enrollment without informed consent obtained in a second time from the patient or surrogate in writing or orally.	950	4 (0.4%)^a^
SAMUKeppra (Navarro et al., 2016)	France	Enrollment with proxy consent or if not available, with authorization from the emergency physician before the enrollment. Patient's post hoc written consent in a second time.	203	47 (23.2%)
Hypothermia for Neuroprotection in Convulsive Status Epilepticus (Legriel et al., 2016)	France	Proxy consent or if not available, authorization from the emergency physician before the enrollment. Patient's post hoc written consent as soon as possible.	270	2 (0.7%)
Randomized Trial of Three Anticonvulsant Medications for Status Epilepticus (Kapur et al., 2019)	USA	Enrollment without informed consent obtained in a second time from relatives in writing.	400	0% (none noted!)
POLAR (Cooper et al., 2018)	Australia, New Zealand, France, Switzerland, Saudi Arabia, and Qatar	Enrollment without informed consent, proxy written informed consent and subsequently from the patient if he/she regained capacity.	511	11 (2.6%)

^a^ 160/1100 (14%) patients not enrolled, as they lacked informed consent.

## 4 DISCUSSION

While research on critically ill patients is needed to improve their prognosis (Luce et al., 2004), it is generally difficult to involve this vulnerable population in research. This assessment of a randomized trial on adults with acute consciousness impairment shows that data were available for analysis in accordance with the EC requirements in more than 90% of enrolled patients. However, ad hoc waivers granted by the EC allowed analysis of more than 1/3 of patients lacking informed consent. It was possible to identify a statement of wishes reporting willingness to participate to clinical research in less than 1/7 of patients (almost never in patients’ charts). Finally, consent was refused or withdrawn in nearly 7% of enrollments, and data had to be discarded.

The time beyond which lack of consent capacity was considered as permanent was preset at 7 (±3) days (EEG interventions were finished on the 3rd day: proxy consents concerned then the use of patients’ data and not authorization to perform EEG). It is interesting to observe that indeed almost 95% of the subjects who regained their judgment capacity recovered it at 4 days or later, which retrospectively corroborates this time point. During follow‐up, investigators repeatedly tried to define the patient's judgment capacity and to obtain post hoc consents: several attempts had to be carried out per‐protocol, but, unfortunately, no details on this procedure were collected.

Initial steps complied with the current Swiss regulatory requirements: for each analyzable patient, we obtained consent from independent physicians. Informed consents were obtained in approximately 2/3 of analyzable patients, more frequently by proxy; this occurred despite the possible stress related to emergency or critical‐care conditions (Azoulay et al., 2005). International and Swiss laws stipulate informed consent as a prerequisite for research (Federal Act on Research involving Human Beings, 2011a; ICH Harmonised Tripartite Guideline E^2016^ (2016), 2016; World Medical Association, 2013). However, in this study, EC allowed waivers in particular situations, and finally informed consent was waived in 36% of analyzable patients, mostly because of intensive care withdrawal or early death. Of relevance, strictly applying the Swiss law, most of these patients would have been excluded from analysis. This large percentage underlines the paramount importance to carefully define these conditions during redaction of the study protocol: without a dedicated authorization to use these data, a considerable patient proportion (mostly reaching the study primary outcome, namely death) would be lost in such a trial, generating a potentially relevant inclusion bias affecting the results. In a British study, only certain hospitals were allowed to waive proxy consent in emergency situations: waiving consents shortened the average time to randomization from 4.4 to 3.2 hr, and increased the average number of participants from 1.5 to 2 per month (Roberts, 2004). Two other studies are consistent with this observation, showing that waiving consents allows enrolling more patients (Annane et al., 2004; Clifton et al., 2002). Additionally, a Dutch study in an intensive‐care setting found that an intervention effect can be significantly lost after excluding patients lacking deferred consent (i.e., consent obtained after enrollment, by patients or proxy) (Jansen et al., 2010).

Current Swiss law may also prove problematic, in this clinical environment, regarding identification of a clear statement of wishes reporting willingness to participate in a clinical trial. This was identified in approximately 14% of enrolled patients, nearly exclusively related by proxy, despite repetitive attempts. Our proportion seems broadly in line with that reported recently in a Swiss emergency department (20%) (Slankamenac et al., 2020). Unfortunately, the number and details of statement of wishes opposing research were not available in our study, since this was an exclusion criterion and was not protocolled. In practice, very often relatives did not know the patient's opinion upon clinical research, and the information was almost never available in medical files. These observations raise the question about the relevance of repeated efforts to look for these wishes, to be balanced against potential benefits of the implementation of an awareness campaign on research (similar to organ transplantation). Further, “signs and symptoms showing patient's opposition” represent in our view vague concepts not applicable in practice.

While the proportion of 6.7% excluded due to consent issues seems relatively small at first glance, it may exert an effect in terms of study results: as refusals occurred only in survivors (data of patients dying early was managed through waivers), mortality increased in the analyzed sample. Moreover, since no analysis of these patients was allowed, we cannot assure that they did not represent a different subgroup in terms of demographics or etiologies, nor assess the reasons for refusal. It seems reasonable to consider this aspect in future studies in similar settings and allow at least partial use of data from these patients, in order to ensure results’ generalizability. In fact, a recent Canadian study involving critically ill patients showed that those with consent refusals were the most severely ill (Tropolovec‐Vranic et al., 2014), while in our study only alive patients could refuse. Rates of refusals or withdrawals appear much lower when formal consent is not required due to general waivers in emergency situations (Kapur et al., 2019), where often the only requirement is to inform the relatives on the opportunity for the patient to oppose the use of data (0.5%; Kapur et al., 2019; Lascarrou et al., 2019), or when informed consent can be obtained orally (1.2%; Nielsen et al., 2013). Conversely, refusals seemed higher (23.2%) in a recent French trial on convulsive SE, in which proxy consent was required (or, if unavailable, an authorization from a physician; Navarro et al., 2016). Globally, in recent studies, the variability of consent refusals appears wide and is probably related to different regulations and study designs.

A recent US assessment focusing on emergency conditions identified 28 studies using consent waivers over the last 2 decades; only 46% of them detailed on its justification (Klein et al., 2018). In the United States, the Department of Health and Human Services issued a Common Rule in 1996, subsequently updated, where recruitment of patients is not able to consent is foreseen in emergency situations (https://www.fda.gov/files/about%20fda/published/Exception‐from‐Informed‐Consent‐Requirements‐for‐Emergency‐Research.pdf; Bauer & Tate, 2020). FDA regulations foresee consent waivers for the emergency use of a test article in determined situations (21CFR 50 and 21CFR56); they also provide for waiver of informed consent for planned emergency research under 21CFR50 (https://www.accessdata.fda.gov/scripts/cdrh/cfdocs/cfcfr/CFRSearch.cfm?CFRPart=50). This has to occur under EC supervision, and participants should have life‐threatening conditions, impaired decisional capacity with no time to obtain surrogate consent, and the research intervention should offer a chance of benefit. Furthermore, the investigator should define a time‐window to look for a proxy or legal representative. The latter point seems similar to the Swiss law, but the window is potentially longer in Switzerland (up to 6 months in our study: consent has to be sought as long as a patient is in the study). Additionally, the need to actively look for a previous statement of wishes and the lack of explicit phrasing regarding possible consent waivers in emergency situations seems peculiar for Switzerland.

Although we analyzed a prospectively collected set of data, the lack of information regarding patients in whom consent was refused represents a major limitation. Additionally, our retrospective analysis prevented addressing further aspects, such as quantifying the time spent for obtaining consents. The sample size was not specifically powered for this analysis (but tailored for identification of mortality differences across EEG intervention groups). Consent capacity was not evaluated quantitatively. Finally, this study is not automatically applicable to a pediatric population, where regulatory requirements may differ significantly, and in places outside Switzerland. We however believe that since Swiss regulations closely follow the Declaration of Helsinki (World Medical Association, 2013) and Good Clinical Practice (ICH Harmonised Tripartite Guideline E^2016^ (2016), 2016), it is reasonable to assume generalizability of our findings.

## 5 CONCLUSION

In our RCT, data from 7% of recruited subjects had to be discarded due to lack of informed consent; this influenced the primary endpoint (mortality). Furthermore, more than 1/3 of recruited subjects, mostly dying early, could be included and analyzed only following specific waivers accorded by the EC. This underscores the importance of such waivers, especially in a clinical context where risk of participation is judged low, and to address reasons of drop‐out due to lack of consents, in order to ensure generalizability of results. In this particular environment, looking for patients’ statements on willingness to participate to research seems to have a low yield, without dedicated public campaigns. Finally, we highlight the efforts to achieve high ethical standards in research with participants unable to consent in emergency setting. Such efforts should be considered when assessing the value of studies, beyond statistical results, particularly when comparing works from different consent strategies and settings.

## CONFLICT OF INTEREST

On behalf of all authors, the corresponding author states that there is no conflict of interest.

## AUTHORS’ CONTRIBUTIONS

Milène Guinchard: contributed to data analysis, drafting of the manuscript, and drafting of the figures. Loane Warpelin‐Decrausaz: contributed to study conception and design, data analysis, and drafting of the manuscript. Kaspar Schindler, Stephan Rüegg, Mauro Oddo, and Vincent Alvarez: contributed to data collection and critical review of the manuscript for intellectual content. Jan, Novy: contributed to data collection, critical review of the manuscript for intellectual content, and drafting of the figures. Andrea O. Rossetti: contributed to data collection, study conception, and design, data analysis, and drafting of the manuscript.

### Peer Review

The peer review history for this article is available at https://publons.com/publon/10.1002/brb3.1965.

## Supporting information

Supplementary Material

## Data Availability

The data that support the findings of this study are available from the corresponding author upon reasonable request.
